# The impact of restricted length of treatment field and anthropometric factors on selection of head and neck cancer patients for treatment on the MR-Linac

**DOI:** 10.1259/bjr.20200023

**Published:** 2020-05-21

**Authors:** Brian Ng-Cheng-Hin, Christopher Nutting, Kate Newbold, Shreerang Bhide, Dualta McQuaid, Alex Dunlop, Kevin Harrington, Kee Howe Wong

**Affiliations:** 1 The Institute of Cancer Research and Royal Marsden NHS Trust, Head and Neck Radiotherapy and Imaging, Sutton, United Kingdom; 2 Joint Department of Physics, The Institute of Cancer Research and Royal Marsden NHS Trust, Sutton, United Kingdom

## Abstract

**Objective:**

This study investigates the impact of a restricted craniocaudal (CC) field length of <20 cm on the selection of head and neck cancer (HNC) patients who can be treated on the MR-Linac using a single isocentre technique. We also assess the effects of anthropometric factors and the neck position on the CC field length.

**Methods:**

110 HNC patients who underwent radical primary or adjuvant radiotherapy were retrospectively analysed. We assessed the proportion of treatment fields with a CC length of <20 cm and the effects of gender, height, hyo-sternal neck length (distance from superior surface of hyoid to sternal notch measured on the coronal reconstruction of the planning CT) and neck position on CC length.

**Results:**

95% of HNC patients had a CC field length <20 cm. Female patients showed a significantly shorter median CC length than male patients in both extended (*p* = 0.0003) and neutral (*p* = 0.008) neck positions. Neck position influenced the median CC length with neutral neck being significantly shorter than extended neck (*p* = 0.0119). Patient height and hyo-sternal neck length showed positive correlation with the CC length, with neck length in neutral position having the strongest correlation (*r* = 0.65, *p* = 0.0001 and *r* = 0.63, *p* < 0.0001, respectively for extended neck; *r* = 0.55, *p* = 0.0070 and *r* = 0.80, *p* < 0.0001, respectively for neutral neck). A hyo-sternal neck length of <14.6 cm predicted a CC length of <20 cm in neutral neck position.

**Conclusion:**

The majority of patients with HNC at the Royal Marsden Hospital have anthropometric features compatible with their being treated on the MR-Linac using a single isocentre technique. The absolute CC field size may vary according to primary tumour site, patient factors and neck position. A hyo-sternal neck length cut-off of 14.6 cm in the neutral neck position can be used as a surrogate marker for suitability of treatment on MR-Linac.

**Advances in knowledge:**

This paper highlights the potential impact of a restricted CC field in HNC patient selection for the MR-Linac treatment. This is the first report to suggest the use of neck length as a surrogate marker for suitability of treatment on the MR-Linac.

## Introduction

The Elekta Unity magnetic resonance-linear accelerator (MR-Linac) is a hybrid system that integrates the imaging capability of a 1.5 T MR scanner (Philips Healthcare, Best, The Netherlands) with a linac (Elekta, AB, Stockholm, Sweden). Anatomical and functional MR imaging can be conveniently acquired at the time of treatment, enabling real-time assessment of intra- and interfraction changes, unlocking the potential for daily plan optimisation and adaptive radiotherapy treatment. However, integrating a linac within a strong magnetic field requires modification of certain components of the MR system^1^ and appreciation of the effects of the magnetic field on the behaviour of charged particles (Lorentz force), which means that the MR-linac has some important differences to a standard linac. As the magnetic field remains active during treatment delivery, scattered secondary electrons can bend back at the air–tissue interfaces (electron return effect) or spiral along the magnetic field (air-electron streaming effect). These electrons can deposit in the skin and lung,^2^ and on surfaces perpendicular to the magnetic field such as the jaw, armpits and arms.^3^ These must be accounted for at the planning and optimising stages to reduce unwanted radiation dose deposition outside of the treatment field.^3,4^

The MR gradient coil is a crucial component located within the MR bore and produces calibrated distortions of the main magnetic field in the *x, y* or *z* axes to enable localisation of the image slices. This coil is physically split to enable a radiation window which limits the maximum field size at the isocentre to 22 and 57 cm in the CC and lateral directions, respectively.^2,5^ Another important difference is that the MR-linac has a static couch and set-up errors are corrected by shifting beam apertures.^6^ A 1 cm margin in all directions has been suggested for plan adaptation to the daily anatomy and set-up errors.^7^ This restricts the maximum radiation field to 20 cm in the CC direction and may influence the selection and the absolute number of head and neck cancer (HNC) patients who can be treated on the MR-Linac using a single isocentre technique.

This study aimed to assess: (a) the effect of a restricted CC field length on the suitability and selection of HNC patients who can be treated on the MR-Linac using a single isocentre technique; (b) the association between CC field length and anthropometric factors such as gender, height, hyo-sternal neck length and treatment position.

## Methods and materials

### Patient selection

This retrospective study included a total of 110 HNC patients who underwent either radical primary or adjuvant (chemo)radiotherapy at the Royal Marsden Hospital between January 2018 and June 2019. The HNC subsites included oropharynx, nasopharynx, hypopharynx, larynx, paranasal sinus, parotid, oral cavity and unknown primary. All patients consented to have their imaging used for research purposes. To investigate the “worst-case” scenario, only patients with a radiation field encompassing both primary site and neck nodal levels were included. All these patients were planned using either intensity modulated radiotherapy (IMRT) or volumetric arc therapy (VMAT).

**Figure 1.**
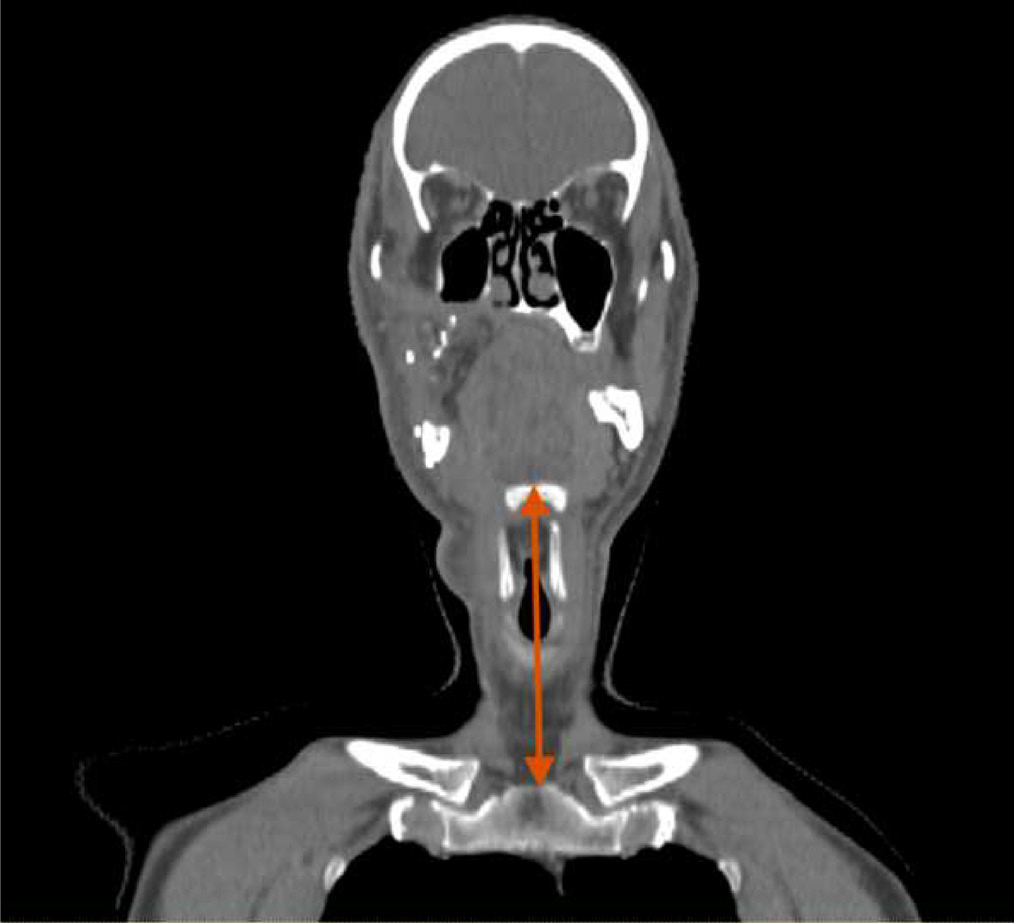
An example of neck length measurement on a coronal CT slice. Neck length was defined as the distance between the most cranial aspects of the hyoid bone and sternal notch (double ended arrow in red).

Baseline characteristics such as gender, height, neck length and TNM staging (American Joint Committee on Cancer seventh edition) were collected. Neck length was defined as the distance (cm) between the superior surface of hyoid to sternal notch measured in the midline on the coronal reconstruction of the radiotherapy planning CT scan (Figure 1).

### Radiotherapy planning image acquisition

**Figure 2.**
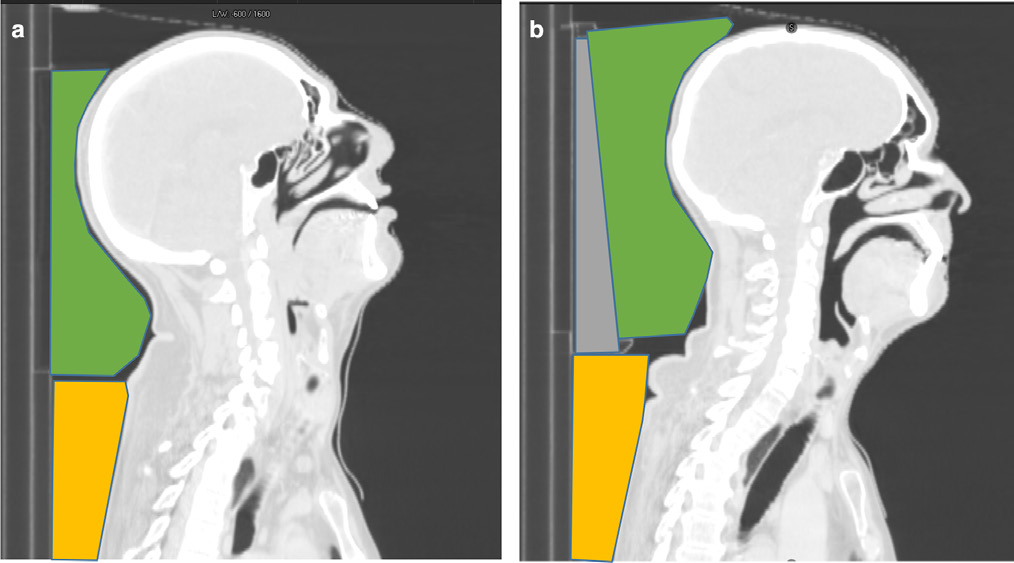
Patient set-up illustrated for extended neck (A) and neutral neck (B) positions on a sagittal CT scan slice. Neck position is altered using a combination of a headrest (green), shoulder wedge (yellow) and wedge (grey).

Patients were immobilised with a custom-made 5-point thermoplastic mask and scanned in the supine position on a large-bore CT scanner (Philips Medical, Cleveland, OH). Scans were acquired in 2 mm slices. At the Royal Marsden Hospital, patients with pharyngeal and laryngeal HNC were scanned and treated in an extended neck position. This originates from the PARSPORT trial where the neck was comfortably extended to help reduce the radiotherapy dose to the oral cavity whilst also sparing the parotid glands.^8,9^ Other HNC sites, such as the oral cavity and paranasal sinuses, were scanned and treated in a neutral neck position. The difference in neck positions is illustrated in Figure 2.

### Target volume delineation and craniocaudal (CC) length measurement

All cases were delineated on the clinical version of Raystation 8.0 (RaySearch Laboratories, Stockholm, Sweden). The target volume delineation was performed according to local and international guidelines^10,11^ for respective tumour sites. There were three dose levels for clinical target volume (CTV): high, intermediate and low dose CTVs corresponded to 65, 60 and 54 Gy in 30 fractions delivered over 42 days. The planning target volume (PTV) was generated using 3 mm isometric expansion of CTV as per our institution’s protocol. The CC field length was derived by measuring the absolute distance between the most cranial and caudal aspects of the PTV.

To investigate the influence of neck position on CC treatment field length, 23 patients with oral cavity cancer and 51 patients with oropharyngeal cancer were selected to represent the neutral and extended neck cohort. To enable comparison, we simulated the longest treatment field for node-positive HNC by delineating standardised CTVs that extended cranially to the skull base to include level VIIb nodes and caudally to level IVa. The CTV was delineated by a single observer (BH) and checked independently by another radiation oncologist specialising in HNC (KHW) for agreement. As per our institution’s protocol, the CTV was expanded by 3 mm isometrically to form the PTV

### Statistical analysis

The data were analysed using Graphpad Prism software (v. 8.2.0; San Diego, CA). The Shapiro–Wilk test was used to test for normality of the data. Mean and median values were reported for parametric and non-parametric data, respectively. The independent *t*-tests and Mann–Whitney were used as parametric and non-parametric tests, respectively. Pearson correlation was used to measure statistical relationships. The strength of the correlation was defined using the following absolute values of r: 0–0.19 as very weak, 0.20–0.39 as weak, 0.40–0.59 as moderate, 0.60–0.79 as strong and 0.80–1.00 as very strong correlation.^12^ Simple linear regression was used to analyse a correlation between CC field length and factors such as patient neck length and height. For this test, logarithmic transformation was used to convert non-parametric data. Differences were statistically significant at two-tailed *p*-values of <0.05.

## Results

Patient and tumour characteristics are summarised in Table 1.

**Table 1.** Patient and tumour characteristics of patients undergoing radical or adjuvant (chemo)radiotherapy

Mean Age (years)	63 (Range 31–85)			
Gender (*n* = 110)	Number of patients	Median craniocaudal field length (cm)	Mean Height (cm)	
Male	76	18 (range, 12 to 25)	176 (SD = 6.1)	
Female	34	14 (range, 11 to 20)	164 (SD = 7.2)	
Tumour Site (*n* = 110)	Number of patients	Craniocaudal field length (cm)		
		Median	Minimum	Maximum
Oropharynx	51	17.6	11.2	20.0
Nasopharynx	3	20.6	18.8	23.0
Hypopharynx	7	15.2	11.8	18.0
Paranasal sinus	3	20.3	20.0	24.6
Unknown Primary	9	17.0	15.2	19.0
Oral Cavity	23	16.8	13.4	19.0
Larynx	13	13.6	11.2	17.8
Parotid	1	18.4	18.4	18.4
Tumour Stage				
T0	9			
T1	18			
T2	37			
T3	25			
T4	21			
Nodal stage				
N0	37			
N1	18			
N2	53			
N3	2			

AJCC, American Joint Committee on Cance;SD, standard deviation.

Staging according to AJCC seventh edition.

### CC treatment field length distribution in the whole HNC population

**Figure 3.**
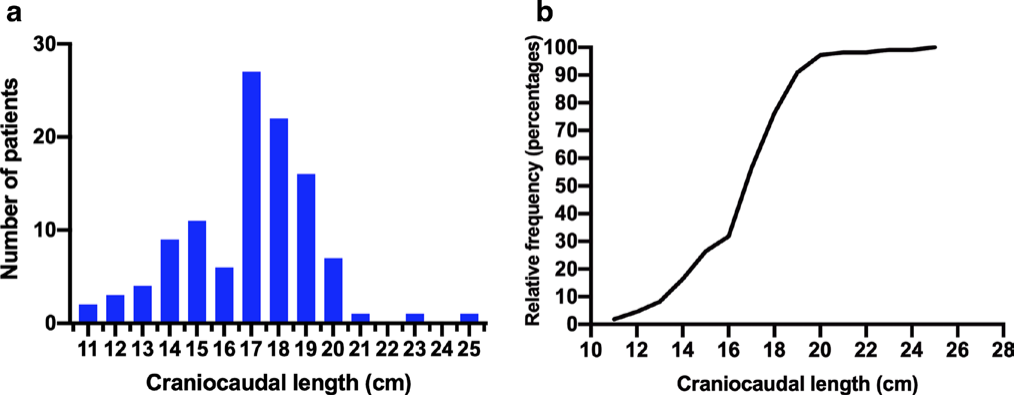
(A) Histogram and (B) cumulative distribution plot illustrating the frequencies of craniocaudal field length in head and neck cancers. *n* = 110.

Overall, 95% of the HNC patients demonstrated a CC field length <20 cm, with the majority (75%) ranging between 15 and 19.9 cm (Figure 3A B). Patients with nasopharyngeal and paranasal HNC had the longest maximum CC field lengths at 23.0 and 24.6 cm, respectively (Table 1).

Six patients had a CC field length of ≥20 cm (Table 2). Their primary sites were nasopharynx (two patients), oropharynx (one patient) and paranasal sinus (three patients). The majority of the patients were male and were taller on average than the overall population (mean height of 177 ± 5.9 cm) *vs* (173 ± 8.6 cm). The median neck length was 13.3 cm (range 10.6–14.6 cm).

**Table 2.** Patient characteristics with a craniocaudal length of ≥20.0 cm. Staging according to American Joint Committee on Cancer (AJCC) seventh edition

Patient	TNM staging	Primary site	Craniocaudal length (cm)	Gender	Height (cm)	Neck Length (cm)	Neck position
**1**	T1 N2b M0	Oropharynx	20.0	M	185	14.6	Extended
**2**	T4b N1 M0	Paranasal	24.6	M	178	12.6	Neutral
**3**	T3 N0 M0	Paranasal	20.3	F	173	13.0	Neutral
**4**	T4a N0 M0	Paranasal	20.0	F	168	10.6	Neutral
**5**	T2 N0 M0	Nasopharyngeal cancer	20.6	M	180	13.6	Extended
**6**	T1 N1 M0	Nasopharyngeal cancer	23.0	M	179	14.4	Extended

AJCC, American Joint Committee on Cancer; F, Female; M, Male.

Staging according to AJCC seventh edition.

### Effect of gender, height and neck length on CC field length in the extended neck position

Female patients had a significantly shorter mean height than male patients (165.0 ± 7.9 cm *vs* a mean height of 177.0 ± 5.5 cm; *p* = 0.0001). Female patients also showed a significantly shorter median CC field length of 17.0 cm (range 11.0–19.0 cm) compared to 18.0 cm (range 16.0 to 20.0 cm) for the male patients (*p* = 0.0003).

Overall, there was a strong positive correlation between patient height and CC field length (*r* = 0.65, *p* = 0.0001) (Supplementary Figure 1). Similarly, neck length also showed a statistically significant strongly positive correlation with CC field length (*r* = 0.63, *p* < 0.0001) (Supplementary Figure 1). As expected, this suggests that the CC length increased with an increase in patient’s height and neck length.

Supplementary Figure 1.

### Effect of neck position on the CC field length

The comparison between neutral and extended neck cohorts is shown in Table 3. Patients scanned in a neutral neck position had a shorter median CC field length than extended neck (15.8 cm (14.8–19.2) *vs* 17.6 cm (13.6–20); *p* = 0.0119). There was no statistical difference in height between the two cohorts (*p* = 0.051), indicating that neck position independently influences the CC field length.

**Table 3.** Comparison of the craniocaudal field length, neck length and patient height for patients scanned in a neutral and extended neck positions

	Craniocaudal field length (cm)			Neck length (cm)			Patient height (cm)	
	Median (cm)	Minimum (cm)	Maximum (cm)	Median (cm)	Minimum (cm)	Maximum (cm)	Mean (cm)	SD (cm)
Neutral neck (*n* = 23)	15.8	14.8	19.2	10.6	9.20	13.6	171	8.87
Extended neck (*n* = 51)	17.6	11.0	20.0	12.0	8.40	15.0	175	1.63

Patient height showed moderate correlation with CC field length in both neutral and extended neck positions (*r* = 0.55, *p* = 0.0070 and *r* = 0.65, *p* < 0.0001, respectively).

In the extended neck position, hyo-sternal neck length showed a strong positive correlation (*r* = 0.63, *p* < 0.0001) with CC field length. Amongst all anthropometric factors, the hyo-sternal neck length showed the strongest positive correlation with CC field length in the neutral neck position, making it the most clinically relevant predictive factor for patient selection suitable for MR-Linac treatment (*r* = 0.80, *p* < 0.0001).

### Proposed height and neck length cut-off values in the neutral neck position

Using simple linear regression, the relationship between neck length (x) and log_(10)_ CC field length (y) was predicted with the following equation:

*y* = 0.02305 *x + 0.9651

This equates to a patient neck length of 14.6 cm predicting a CC field length of 20 cm in the neutral neck position.

Similarly, the relationship between patient height (x) and log_(10)_ CC field length (y) was predicted with the following equation:

*y* = 0.002298*x + 0.8271

This equates to a patient height of 206 cm predicting a craniocaudal field length of 20 cm in the neutral neck position.

## Discussion

The MR-Linac has the potential to deliver truly personalised adaptive radiotherapy for HNC. However, the CC field restriction imposed by a modified MR coil means that not all HNC patients will be suitable for treatment using a single isocentre. First, this analysis shows that the majority of the HNC patients treated at the Royal Marsden Hospital would have a treatment field deliverable by the MR-Linac using a single isocentre, irrespective of the treatment neck position. However, cancers originating from certain subsites such as the nasopharynx and paranasal sinus may not be suitable due to the additional cranial extension of target volumes. For example, the inferior half of the sphenoid sinus needs to be included in the low dose CTV for T1-2 nasopharyngeal cancer and the whole sphenoid sinus if T3-4.^11^ In this study, no paranasal cancer patients and only one of three nasopharyngeal cancer patients had a treatment field length of <20 cm. Recent international delineation guidelines for nasopharyngeal cancer suggest that the lymph nodal levels IV and Vb can be omitted from the low-dose CTV in patients with lymph node-negative neck. Using these guidelines, the lymph node-negative nasopharyngeal cancer patient (Patient 5 from Table 2) would have a craniocaudal field length of 16.4 cm and have a treatment field size suitable for treatment in the MR-Linac. This differs from our institution’s delineation guideline as we include the level IVa lymph nodes in this group of patients. Therefore, the use of these consensus guidelines may make it is possible to treat early stage nasopharyngeal cancers (T1-2 N0 M0) on the MR-L using a single isocentre. A single oropharyngeal cancer patient had a treatment field length of 20 cm. Review of the treatment field showed no delineation deviations. A likely explanation is that this patient’s extended neck position contributed to a longer treatment field, as indicated by our results.

In this study, the lower neck levels IVb (medial supraclavicular) and Vc (lateral supraclavicular lymph nodes) were not included. Nodal level IVb would extend the CC field length caudally to the cranial edge of the sternal manubrium. Including nodal level Vc would not change the CC field length the caudal border of level Vc corresponds to the border of IVa. These nodal levels would be included if involved or at high-risk of harbouring metastatic disease in cases such as nasopharyngeal, hypopharyngeal, subglottic laryngeal and thyroid cancers. Thus, any lower neck treatment requiring level IVb treatment would increase the treatment field size and be difficult to treat on the MR-Linac using a single-isocentre.

The result of this study is based on a maximum CC size of 20 cm due to an isotropic margin of 1 cm. This is a conservative margin which may be reduced further with more clinical experience on the MR-Linac. Reducing the margin would increase the treatable CC field size and increase the number of eligible patients. In fact, a margin reduction to 5 mm has been reported to increase the number of eligible patients by 10%.^7^ However, this remains a topic for further research.

Second, we investigated the impact of anthropometric factors such as height, neck length and position on CC field length that simulated the treatment field of node-positive HNC. Our study demonstrated that hyo-sternal neck length showed a very strong correlation with CC field length in the neutral neck position and was the best predictor of field length in this group. Patient’s height showed a weaker correlation with CC field length and this could be explained by a change in patient height not being in proportion to a change in the patient’s neck length. Other clinically measurable anthropometric factors such as the percutaneous lengths of the ulna^13^ and tibia^14^ have been reported to be predictors of a patient’s stature. However, these measurements were not readily available for correlation with the CC field length and this analysis was beyond the scope of this study.

We showed that female patients had a significantly shorter CC treatment field length compared to male patients, irrespective of neck position and this may be explained by their overall shorter stature. In keeping with this, Vasavada et al demonstrated that females necks are 9–16% smaller than their male counterparts.^15^ Although gender may influence treatment field length, this may not be as influential in tumours that extend cranially. This is illustrated by the two female patients with paranasal cancers had CC treatment field lengths that exceeded the MR-Linac treatment length.

Third, our results suggest that the neck position influences the CC field length. Patients scanned in the neutral neck position demonstrated a smaller median CC field length compared to patients scanned in the extended neck position. The first HNC patient has been treated on the MR-Linac at the Royal Marsden Hospital, with each treatment session lasting up to 40 min. Therefore, a neutral neck position may be preferable to maximise the number of patients eligible for treatment on the MR-Linac and, from experience, help with comfort and tolerance of treatment. With increased IMRT planning experience, an extended neck position is no longer crucial in reducing doses to the oral cavity. Techniques such as using specific dose constraints to the oral cavity may be used.^16^

As we intend to treat patients in the neutral neck position, we derived cut-offs for neck length and patient height to act as surrogate markers for patient selection. These cut-offs were not tested in the overall HNC cohort as these patients consisted of a combination of patients scanned in the extended and neutral neck positions. The upper limits of a neck length of 14.6 cm and a height of 206 cm should be validated in larger studies.

This study has a few limitations. Some subtypes of HNC in this study were under represented with only a small number of patients which prevents us from making concrete conclusions on their suitability. However, patients with nasopharyngeal and paranasal cancers will usually need a longer treatment field that encompasses a target beyond the nodal levels cranially. This means that it is likely that these subtypes will have a treatment field that is not currently treatable on the MR-Linac. This is likely to change with the development of dual isocentre treatment techniques. The lack of matched-controls and a relatively small number of female patients in our analysis means that there may be unaccounted confounding factors that may have affected some of our results. For the neck position analysis, we considered using the diagnostic CT of the patients treated in the extended neck position as this are acquired in a “neutral neck” position. However, we felt that the radiotherapy CT scans of oral cavity patients would be more representative of a “neutral neck” treatment position in view of the immobilisation equipments used.

Despite these limitations, the results of this study reflect those of a study by Chuter et al, who reported that the majority (86%) of their HNC patients would be treatable with a 1 cm adaptive CC margin.^7^ The authors concluded that 75% of their oropharyngeal cancers and 30% of their nasopharyngeal cancer patients would be treatable.^7^ We have reported a larger proportion of patients eligible for treatment on the MR-Linac and the differences may be related to differences in delineation protocols. Our results reflect target delineation according to international guidelines and are, therefore, likely to be applicable to other institutions. To our knowledge, this is the first study to demonstrate a very strong correlation between patient neck length with the CC field length. Currently, a patient’s CC field length is assessed for MR-Linac treatment suitability following review of the radiotherapy planning CT or MR scans. Patient neck length could be used in clinic for patient selection at an earlier stage of the planning process.

## Conclusion

Our results show that the majority of head and neck cancers at the Royal Marsden Hospital have a treatment field that is achievable on the MR-Linac using a single isocentre technique. Primary tumour sites such as nasopharyngeal cancers with significant intracranial extension or paranasal cancers requiring nodal irradiation may not be suitable for treatment on MR-Linac. Our study proposes that a hyo-sternal neck length cut-off of 14.6 cm in the neutral neck position could be used as a surrogate marker for suitability of treatment on MR-Linac and patients at the Royal Marsden Hospital will be treated in a neutral neck position unless there is significant dose distribution benefit from neck extension.
