# Supplementary material for: The impact of restricted length of treatment field and anthropometric factors on selection of head and neck cancer patients for treatment on the MR-Linac
Source: Br J Radiol. 2020 May 21;93:20200023. doi: 10.1259/bjr.20200023 (PMC7336067; doi:10.1259/bjr.20200023)

**Supplementary Figure Captions**

Figure 1. Patients scanned in the extended neck position, n = 51. A) Correlation between patient height and craniocaudal field length. B) Correlation between patient neck length and craniocaudal field length


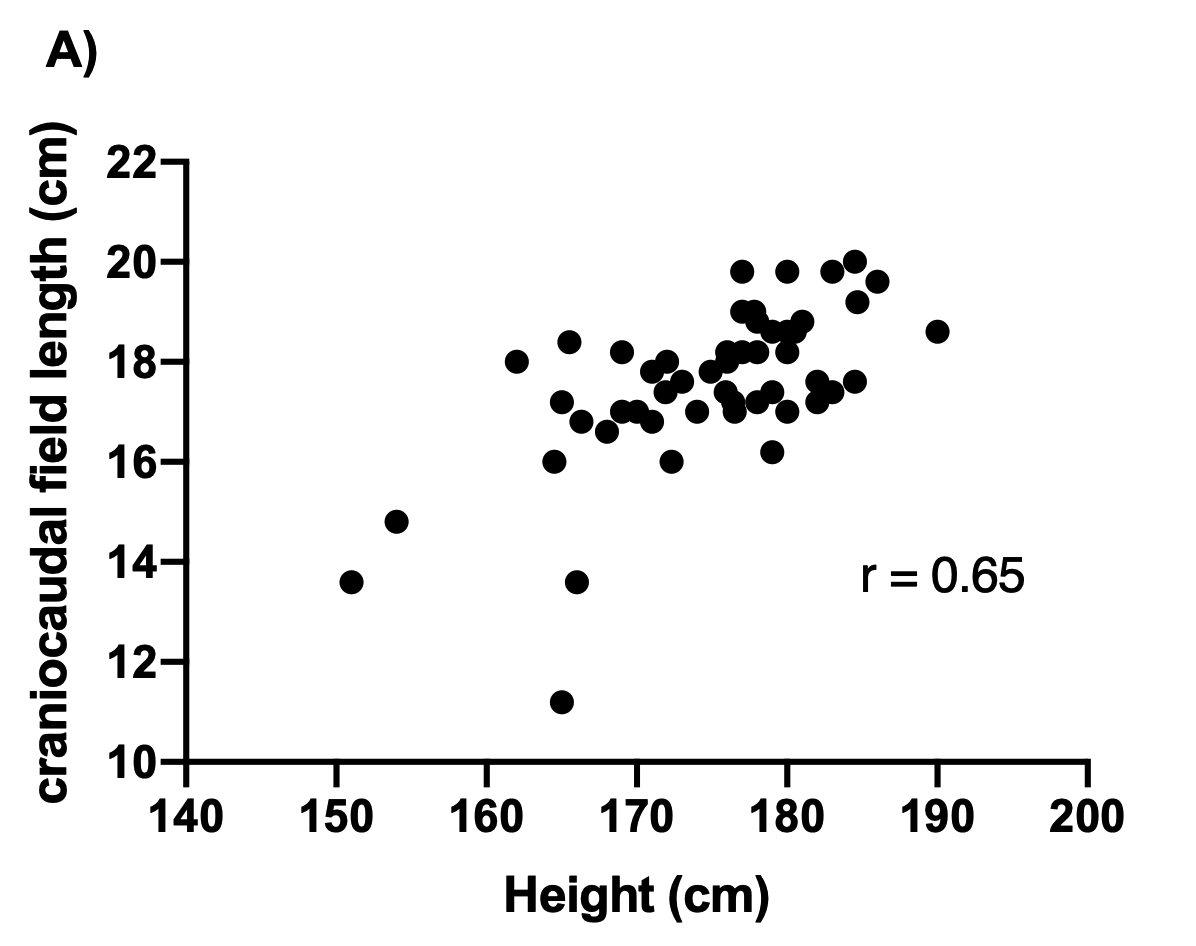

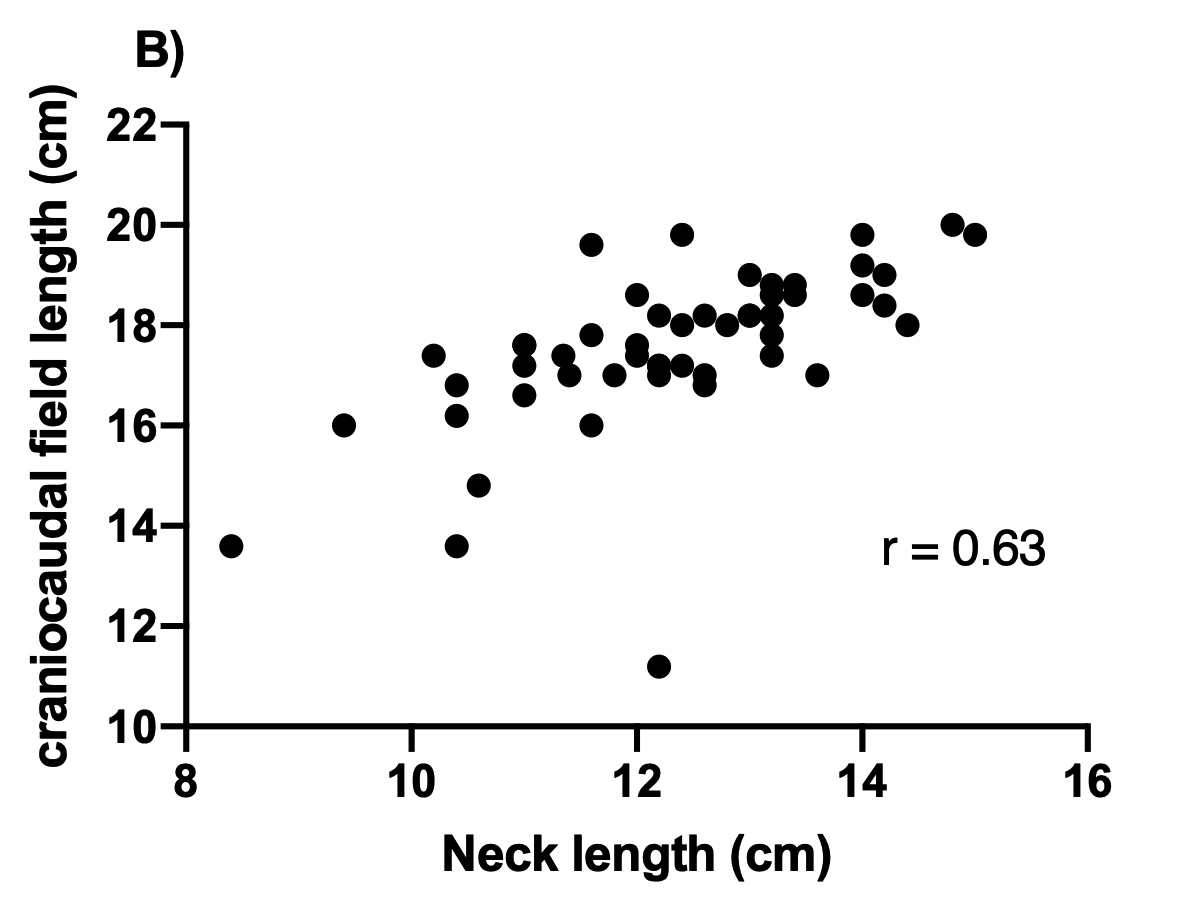

Supplement: Supplementary Figure 1. [file bjr.20200023.suppl-01.docx]
